# IUC22021-72 The inside study: innovative preclinical models for precision medicine in advanced prostate cancer

**DOI:** 10.1093/oncolo/oyaf248.015

**Published:** 2025-08-29

**Authors:** A Opattova, L Bizzozero, S E Bellomo, L Tonelli, E Bollito, F Porpiglia, C Fiori, P Rescigno, C Marchiò, S Arena

**Affiliations:** Candiolo Cancer Institute, FPO–IRCCS, Candiolo TO, Italy; Candiolo Cancer Institute, FPO–IRCCS, Candiolo TO, Italy; Candiolo Cancer Institute, FPO–IRCCS, Candiolo TO, Italy; Candiolo Cancer Institute, FPO–IRCCS, Candiolo TO, Italy; San Luigi Gonzaga University Hospital, Orbassano TO, Italy; San Luigi Gonzaga University Hospital, Orbassano TO, Italy; Department of Oncology, University of Turin, Candiolo TO, Italy; San Luigi Gonzaga University Hospital, Orbassano TO, Italy; Department of Oncology, University of Turin, Candiolo TO, Italy; Candiolo Cancer Institute, FPO–IRCCS, Candiolo TO, Italy and Translational and Clinical Research Institute, Centre for Cancer, Newcastle University, Newcastle upon Tyne, UK—Italy; Candiolo Cancer Institute, FPO–IRCCS, Candiolo TO, Italy and Department of Medical Sciences, University of Turin, Turin, Italy; Candiolo Cancer Institute, FPO–IRCCS, Candiolo TO, Italy and Department of Oncology, University of Turin, Candiolo TO, Italy

## Abstract

**Background:**

Despite progress in prostate cancer (PC) therapies, treatment resistance and low response rates remain major clinical challenges. PC is genomically heterogeneous, with DNA damage repair (DDR) gene mutations present in 10%-19% of primary cases and up to 27% in metastatic castration-resistant PC (mCRPC). While BRCA mutations predict PARP inhibitor (PARPi) response in mCRPC, their role in high-risk, locally advanced PC is unclear, and the impact of non-BRCA DDR alterations is poorly understood. In the era of precision medicine, there is a need for experimental models that faithfully represent tumor heterogeneity. Patient-derived 3D models are emerging as valuable tools to address this gap. This study aims to identify novel actionable vulnerabilities in DDR pathways and assess the clinical impact of DDR alterations beyond BRCA in high-risk advanced PC.

**Methods:**

The INSIDE study is a prospective trial led by the Candiolo Cancer Institute, FPO-IRCCS, with San Luigi Hospital, focusing on DDR gene mutations in high-risk advanced PC. As of March 2025, 68 patients have been enrolled. Tumor samples, together with blood samples, are collected to generate patient-derived organoids (PC-PDOs) for histological, genomic, and pharmacological profiling. Whole-exome sequencing (WES) and TruSight Oncology 500 HRD (TSO500-HRD) are used to analyze genomic variability and genomic instability scores (GIS), offering insights into DDR-related therapeutic targets.

**Results:**

We developed a pipeline to establish PC-PDOs from tumor tissue, achieving a 77% derivation success rate. Notably, 18% of these organoids were suitable for pharmacological screening, an achievement rarely demonstrated for models directly derived from patient tissue, underscoring the translational value of our approach. Preliminary findings suggest DDR effectors such as DNA-PK, ATM, and ATR as promising pharmacological targets. Using TSO500-HRD, GIS was assessed in both patient samples and PDOs, revealing potential sensitivity to PARPi in cases beyond BRCA mutations, including specific missense variants in homologous recombination genes.

**Conclusions:**

The INSIDE study confirms the feasibility of using PDOs to model DDR alterations in high-risk advanced PC. These findings support the integration of genomic and pharmacological data to guide precision therapies, underlining the importance of comprehensive DDR profiling to expand treatment options for PC patients.

